# Echocardiography may help detect pulmonary vasculopathy in the early stages of pulmonary artery hypertension associated with systemic sclerosis

**DOI:** 10.1186/1476-7120-8-25

**Published:** 2010-07-05

**Authors:** Walter Serra, Alfredo Chetta, Daniele Santilli, Flavio Mozzani, Pier Paolo Dall'Aglio, Dario Olivieri, Maria Alberta Cattabiani, Diego Ardissino, Tiziano Gherli

**Affiliations:** 1 Cardiopulmonary Dept., Cardiology Unit, University Hospital, Parma, Italy; 2 Cardiopulmonary Dept., Pneumology Unit, University Hospital, Parma, Italy; 3 Immunology Dept., Rheumatology Unit, University Hospital, Parma, Italy

## Abstract

**Background:**

Pulmonary arterial hypertension (PAH) in patients with systemic sclerosis is associated with a poor prognosis, but this can be improved by early disease detection. Abnormal pulmonary and cardiac function can be detected early by means of echocardiography, whereas right heart catheterization is usually performed later.

**Objectives:**

The purpose of this prospective study was to detect early the presence of pulmonary artery vasculopathy in patients with verified systemic sclerosis without significant pulmonary fibrosis, normal lung volumes and a mildly reduced lung diffusion capacity of carbon monoxide (DLCO).

**Methods:**

Nineteen consecutive female NYHA class I-II patients with scleroderma and a PAPs of < 35 mm/Hg measured by echocardiography, were enrolled between September 2007 and September 2009. They had a mean age of 51 ± 13 years, body mass index of 25 ± 5 kg/m^2^). They all underwent complete Doppler echocardiography, CPET, a pulmonary ventilation test (carbon monoxide lung diffusion, DLCO), HRCT. To investigate PAH by means of complete resting Doppler echocardiography estimates of systolic pulmonary artery pressure (PAPs) derived from tr icuspid regurgitation, mean PAP derived from pulmonary regurgitation, pulmonary vessel resistance (PVR) derived from the acceleration time of the pulmonary outflow tract (ACTpo), and right ventricular function derived from tricuspid annular plane systolic excursion (TAPSE). Right heart catheterisation was conducted only, if pulmonary hypertension was suggested by echocardiography and an abnormal ventilator test.

The data are given as mean values ± SD, unless otherwise stated. The correlations between the variables were analysed using Pearson's *r *coefficient, and the predictive value of the variables was calculated using linear regression analysis. A p value of > 0.05 was considered significant.

**Results:**

Right heart catheterization detected PAH in 15/19 patients; mean PAP was 30.5 mm/Hg and RVP 3.6 UW. Coronary angiography of the patients aged more than 55 years showed some evidence of significant coronary artery disease. Echocardiography showed high systolic PAP values (46 ± 8 mmHg), whereas right ventricular function was normal (TAPSE 23 ± 3 mm), and in line with the NYHA class. ACTpo was reduced in the patients with a systolic PAP of < 46 mm/Hg (p > 0.001) and positively correlated with DLCO (p > 0.001) and the hemodynamic data.

There was a good correlation between ACTpo and PVR (hemodynamic data) (r = -0615; p > 0.01).

**Conclusions:**

Although they need to be confirmed by studies of larger series of patients, our findings suggest that, in comparison with hemodynamic data, non-invasive echocardiographic measurements are an excellent means of identifying early-stage PAH.

## Introduction

Systemic sclerosis is a chronic disease essentially due to microvascular abnormalities, antibodies and a fibroblast production that leads to excessive collagen levels, and skin and viscera fibrosis[1-3].

The most recent data show that the prevalence of systemic sclerosis from 1977 to 1980 was 12.6-25/100,000, with an incidence of 0.06-1.9/100,000. The recorded mean age of the patients at the time of onset is between 45 and 65 years, this data does not reflect the real age as the symptoms may appear many years before the first specialist assessment. The main pulmonary complications are pulmonary fibrosis and pulmonary arterial hypertension (PAH): interstitial lung disease is observed in 70% of the patients with diffused systemic sclerosis, and pulmonary hypertension in 35% of those with limited systemic sclerosis[4,5]. The patients have no symptoms at rest during the initial stages of the disease, but soon develop slight dyspnea during physical exercise or rest; some may also experience chest pain, asthenia and loss of conscience under stress.

As pulmonary hypertension in systemic sclerosis may not only be due to PAH, but also to interstitial lung disease and cardiac involvement[6-9], it is very important to classify it adequately[10,11] in order to determine the best treatment. According to the 2009 guidelines[12], PAH describes an increase in mean pulmony artery pressure (PAPm) to more than 25 mmHg. Echocardiography and respiratory function testing are recommended every year, even if the patient is asymptomatic, and right heart catheterisation is usually performed in late stages.

The published frequencies of PAH in systemic sclerosis vary widely (from 5% to 35%), depending on the study population, and diagnostic methods (cardiac echo-Doppler or right heart catheterisation) and criteria. Echocardiography plays a central role in the non-invasive screening and management of patients with suspected PAH as it guarantees the early identification and treatment of a condition that is usually diagnosed at least two or three years after its onset[13-15].

PAH and interstitial lung disease are the leading causes of death in systemic sclerosis and, as their early detection and treatment should improve morbidity and mortality[16,17]. The aim of this prospective study was to detect the presence of pulmonary artery vasculopathy early in patients with verified systemic sclerosis and normal lung volumes, no significant pulmonary fibrosis, and mildly reduced lung carbon monoxide diffusing capacity (DLCO) by: 1) evaluating resting pulmonary function at rest, and maximum and sub-maximum exercise capacity; 2) detecting systolic pulmonary artery pressure (PAPs) values of < 35 mmHg using thoracic echocardiography; and 3) investigating the relationships between PAP and the acceleration time of the pulmonary outflow tract (ACTpo), right ventricular function (tricuspid annular plane systolic excursion; TAPSE), and right heart catheterisation (PAPs, PAPm and pulmonary vessel resistance [PVR]).

## Patients and Methods

19 female patients, aged between 28 and 75 (51 ± 13); BMI (Kg/m2) 25 ± 5, NYHA class I^-II^ diagnosed with Systemic Sclerosis were enrolled between 2007 and 2009 in collaboration with the Rheumatic Department of the University Hospital of Parma (EULAR Scleroderma Trials criteria)[18].

All patients had standard pulmonary function tests, including FVC and DLCO; auto-antibodies (ANA) including anti-centromere, anti-Scl-70 were performed as available from commercial laboratories.

A chest radiograph and a high resolution CT scan (HCRT) were performed to select patients without significant pulmonary fibrosis.

Patients without significant pulmonary fibrosis with reduced lung volumes, left heart dysfunction and significant valvular disease were enrolled. [see Additional file 1]

***ECG***: rhythm, heart rate and strain in the right ventricle

### Doppler echocardiography

The investigated echocardiographic parameters were PAPs (derived from a tricuspid regurgitation flow value of < 2.8 m/s), ACTpo (normal value < 130 msc), PVR (using the equation: PVR = TRV/TVI rvot × 10+0.16)[19], inferior vena cava flow, diameter and inspiratory changes (normal value > 18 mm with respiratory collapse < 50%), and Doppler tissue imaging (DTI) of the tricuspid annulus, which were evaluated using a Phillips IE33 echocardiograph equipped with 2.5 and 3.5 MHz electronic transducers, harmonic imaging and DTI.

### Respiratory function

Respiratory function was tested in accordance with international recommendations using a pneumotachograph and plethysmographic cabin connected to a computer for data analysis (Vmax 22 and 6200, Sensor Medics, Yorba Linda, CA)[20,21].

The analysed parameters were total lung capacity (TLC), volume residue (VR), forced expiratory volume in one second (FEV_1_), and the FEV_1_/FVC ratio. DLCO was calculated using the single breath method, and the test was only considered valid when the inspiratory volume was the same as at least 90% of FVC. Each variable was measured at least three times. The theoretical lung volume and DLCO values were obtained using the Quanjer and Cotes regression equations[22].

### Six-minute walking test

The six-minute walking test (6MWT) consists of walking along a 30-metre corridor for the longest distance possible in six minutes using a standard protocol; as the test is symptom-limited, the patients are allowed to stop and resume walking when they wish. Before and after the test, the patients' vital parameters were recorded and the patients complete a visual analogue scale (VAS) quantifying the degree of dispnea. Oxygen saturation (SPO_2_, expressed as a percentage) and heart rate (HR, expressed as beats per minute [bpm]) were continuously monitored and recorded every 10 seconds from five minutes before the walk to five minutes after returning to baseline values using a portable pulsometer (Model 920 M, Healthdyne, Marietta, GA). To allow for the learning effect, a pause of 60 minutes was allowed between each test.

### Cardiopulmonary exercise test

A cardiopulmonary exercise test CPET) was performed following a standardised procedure (ATS/ACCP Statement, 2003)[23]. After the oxygen and carbon dioxide analysers and the flow mass sensor had been calibrated, the study patients were asked to sit on an electromagnetically braked cycle ergometer (Corival PB, Lobe BV, Groningen, The Netherlands) for a 3-minute rest period, with the saddle adjusted to avoid maximum extension of the knee. Exercising began with a 3-minute warm-up period at 0 watts, followed by a progressively increasing ramp protocol of 5-15 watts/min (depending on the anthropometric data and the patients' degree of functional impairment) for a period of 8-12 minutes. The patients were required to maintain a pedalling frequency of 60 rpm, as indicated by a digital display on the monitor of the ergometer.

During the test, breath-by-breath VO_2 _(mL/min), carbon dioxide production (VCO_2_, mL/min) and minute ventilation (VE, L/min) were collected (Vmax 229, Sensor Medics, Yorba Linda, CA), the patients were continuously monitored by means of a 12-lead electrocardiogram (Corina, GE Medical Systems IT Inc., Milwaukee, WI) and pulse oximeter (Pulse Oximeter 8600, Nonin Medical Inc., MN), and blood pressure was measured at two-minute intervals. Resting end-tidal CO_2 _pressure (rest PETCO_2_, mm Hg) was recorded as the mean value measured during the 3-minute rest period, and peak VO_2 _as the mean value during the last 20 seconds of the test (expressed in mL/min and mL/kg/min). Ventilatory response to exercise was calculated as a linear regression function by plotting VE against VCO_2 _every 10 seconds of exercise (VE/VCO_2 _slope).

### Right heart catheterisation

The patients aged more than 55 years underwent femoral right heart catheterisation using a 7F catheter thermo dilution, to evaluate systolic, mean and capillary pulmonary pressure and calculate the trans-pulmonary gradient (GTP = PAPm/CO), PVR (dynes.scm-5 = GTP/CO × 80) and cardiac output (CO). PAH was defined as a PAPm of < 25 mm/Hg and Pulmonary Capillary Wedge Pressure (PCWP) of > 15 mm/Hg. Measuring the pressure of the pulmonary occlusion makes it possible to identify the pulmonary pressure site and allows the diagnosis of the pulmonary hypertension site (pre-/post-capillary).

### Statistical analysis

The primary objectives were to assess the associations between DLCO% at rest and PAP, and between the echocardiographic and right heart catheterisation measurements of PVR. All of the data are given as mean values ± SD unless otherwise stated. Pearson's correlation coefficient and Bland-Altman analysis were used to compare PAPs, ACTpo and TAPSE with DLCO, VO2max%, VE/VCO2, O2 pulse, NYHA class and PVR. Stepwise forward multiple regression analysis allowed the weighting of the independent effects of the potential determinants on an independent variable. The null hypothesis was rejected when p was > 0.05.

ACTpo was measured by two echocardiographers blinded to the clinical data in order to assess interobserver variability.

## Results

Seventeen of the 19 patients had limited cutaneous systemic sclerosis, and two an overlap syndrome including scleroderma.

### Pulmonary function data

The patients were selected because the results of respiratory function testing at rest (FVC/DLCO) aroused a suspicion of pulmonary vasculopathy (Tab 1). Flow, volume were normal, but not DLCO (71% of the theoretical value) and FV/DLCO (1.5). The distance covered in the 6MWT was normal (424 ± 102 m) (Tab 2). During the cardiopulmonary test, there was a moderate reduction in exercise capacity (VO2max% theoretical 68 ± 14%), a reduction in oxygen pulse (8.1 ± 1.7 ml/beat), and a reduction in the equivalent CO2 ventilators (VE/VCO2 slope 38 ± 12) (Tab 3).

**Table 1:** The functional respiratory values at rest (flow, volume, MIP and MEP) were normal except DLCO (71% ± theoretical) and FV/DLCO (1,5)

DATA	Mean ± SD
Age (y ±)	*54 ± 11*

BMI (Kg/m^2^)	*25 ± 5*

FEV_1 _(L)	*2,47 ± 0,60*

FEV_1 _(% theoretical)	*106 ± 26*

FEV_1_/SVC (%)	*81 ± 3*

TLC (% theoretical)	*97 ± 20*

FVC (% theoretical)	*102 ± 18*

DLCO (% theoretical)	*71 ± 18*

FVC/DLCO	*1,5 ± 0,4*

**Table 2:** The 6MWT showed a normal range test (424 +-102 m)

Parameter	Mean ± SD
Distance (meter)	*424 ± 102*

Distance(% theoretical)	*122 ± 12*

Oxigen Hemoglobinic Desaturation (%)	*2 ± 3*

**Table 3:** CPET showed: VO2 max % theoretical (68 ± 14), a reduction in the oxygen pulse (8,1 ± 1,7) and the equivalent CO2 ventilators (VE/VCO2 slope 38 ± 12)

Parameter	Mean ± SD
V'O_2_max (ml/kg/min)	*19,9 ± 7,1 *

V'O_2_max (L/min)	*1,18 ± 0,34 *

V'O_2_max (% theoretical)	*63 ± 14 *

V'O_2_@AT (l/min)	*0,90 ± 0,26 *

V'O_2_@AT (ml/kg/min)	*14,6 ± 5,5 *

Watts	*75 ± 34 *

(% theoretical)	*77 ± 20 *

V'O_2_/Watts	*7,2 ± 2,7 *

HR max (bmp)	*146 ± 24 *

HR (%)	*88 ± 13 *

O_2 pulse _(ml/bpm)	*8,1 ± 1,7 *

HR/VO_2 _	*66 ± 20 *

VE max (%)	*50 ± 20 *

VE/VCO_2 _(*slope*)	*38 ± 12 *

### Echocardiography

Systolic pulmonary pressure was high (46 ± 8 mmHg), but right ventricular function was normal (TAPSE 23 ± 3), and in line with the patients' NYHA class (Tab. 4).

**Table 4:** Echo data showed high pulmonary systolic pressure values (46 ± 8 mmHg), while the right ventricular function was normal (TAPSE 23 ± 3)

Parameter	Mean ± SD
NYHA (I-IV)	II (I-II)*

PAPs (mm Hg)	*46 ± 8*

TAPSE (mm)	*23 ± 3*

ACTpo (msec)	*117 ± 6*

PVR (WU)	*3.31 ± 2*

In the patients with high PAPs, echocardiography showed a reduction in acceleration time on the pulmonary flow velocity curve (ACTpo 121 msc) (p > 0.001) that corresponded with the diffusion capacity at rest (DLCO%).

Mean ACTpo and PVR were respectively 117 msc and 3.31 UW.

There was a significant correlation between PAPs and NYHA class (r = 069; p > 0.05), between TAPSE and NYHA class (r = -0.47; p > 0.05), and between DLCO and peak VO2 (p > 0.05), and a good correlation between ACTpo and NYHA class (r = - 0.71; p > 0.001). [see Additional file 2]

### Right heart catheterisation

Right heart catheterisation detected PAH in 15/19 patients, and one patient had veno-occlusive pulmonary hypertension; PAPm was 30.5 mmHg and PVR 3.6 UW.

**Figure 1:**
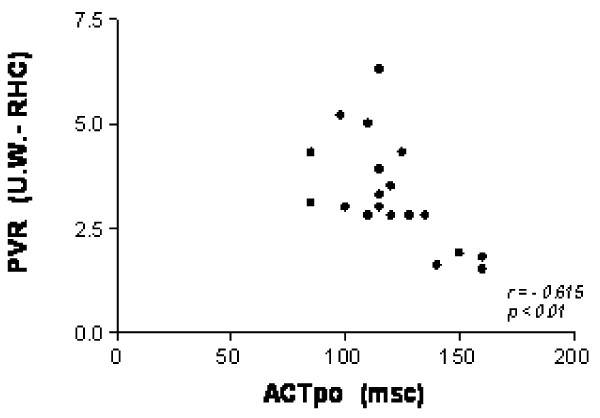
**Correlation between ACTpo and PVR (RHC)**.

There was a good correlation between ACTpo and PVR (r = - 0615; p > 0.01) (Fig 1).

Coronary angiography (carried out in the patients aged < 55 years) did not reveal any signs of significant coronary artery disease.

## Discussion

Pulmonary artery hypertension is the most frequent cause of death in scleroderma patients, and its early identification is the best means of improving survival.

By allowing a non-invasive evaluation of the right atrium ventricular gradient, post-systolic tricuspid annular movement (TAPSE) and the acceleration time of right outflow (ACTpo, an indirect marker of pulmonary vascular resistance), echocardiography reveals the presence of pulmonary hypertension even in patients in a less advanced functional class. The data obtained by means of invasive right heart catheterisation confirmed our echocardiographic findings, which overestimated three cases with tricuspid gradient values of > 3 m/sec [24-26].

Echocardiographic measurements of the tricuspid gradient are accurate but may lead to overestimated pulmonary pressures or, less frequently, underestimates due to an incorrectly aligned regurgitated jet. Valchiery *et al. *found a good correlation between invasive and non-invasive (echocardiographic) measurements of PAPs (r = 0.57, p > 0.001), but other authors have found discrepancies in 35% of cases[27]. The discrepancies are possibly due to factors such as inexperience with the technique, an incorrectly aligned regurgitated jet (which can be better defined using contrast medium), or incorrect right atria pressure, which can be better evaluated using the diameter and the collapse of the inferior vena cava during a breath in.

According to the 2009 guidelines[12], PAH should not be diagnosed by means of echocardiography alone. However, as it detected pulmonary vasculopathy early on the basis of the ACTpo, TAPSE and PAPs findings, we believe that the echocardiographic estimate of PVR should become a part of a standard examination. Three-dimensional echocardiography or cardiac magnetic resonance may be useful in evaluating right ventricular function, but neither were used in this study.

Echocardiography and CPET are both essential for screening patients at risk of developing PAH, which in any case needs to be confirmed by catheterisation, which also provides prognostic data that are useful for therapeutic purpose[26-29].

## Conclusions

The alterations in cardiorespiratory function described above represent initial signs of pulmonary and cardiac vessel impairment in patients with systemic sclerosis. Echocardiographic findings non-invasively reveal the presence of increased pulmonary pressure and pulmonary vascular resistance, morphological changes, and functional abnormalities in right ventricular parameters, which cannot be identified by means of catheterisation. The early recognition of vascular involvement together with a multidisciplinary approach to the illness should lead to better therapeutic results[30,31].

### Study limitations

The main limitation of this study is the small number of patients, and so the findings need to be confirmed in larger series. Misalignment due to the equalisation of pressure between the right atrium and ventricle may lead to underestimated echocardiographic calculations of PAPs and PVR, but we found a good level of inter-observer agreement concerning tricuspid regurgitation velocity and pulmonary outflow acceleration time (p > 0.5).

## Abbreviations

ACTPO: Pulmonary outflow acceleration time; BR: Breath reserve (MVV-VEmax); CO: Cardiac Output; CPET: Cardio Pulmonary Exercise Test; DLCO: Carbon monoxide lung diffusion.; FEV_1_: Forced expiratory volume in 1 second; FVC: Forced vital capacity; GTP: Transpulmonary gradient (PAPm/CO); MVV: Maximum Voluntary Ventilation; NYHA: New York Heart Association; O_2_Pulse: Oxygen Pulse (VO2/HR); PAH: Pulmonary Arterial Hypertension; PAPm: Pulmonary Artery Pressure mean; PCWP: Pulmonary Capillary Wedge Pressure; PVR: Pulmonary Vessel Resistance; RV: Residual Volume; SS: Systemic Sclerosis; TAPSE: Tricuspid annular plane systolic excursion; TLC: Total Lung Capacity; VAS: -Visual analogic Scale; VO2: Oxygen Consumption; VCO2: Carbon Dioxide production; VE/VCO2 slope: CO2 Ventilatory equivalent slope; VE/VO2 slope: O2 Ventilatory equivalent slope; 6MWT: 6 Minute Walking Test.

## Competing interests

The authors declare that they have no competing interests.

## Authors' contributions

WS conceived and carried out the study and drafted the manuscript. AC participated in the design of the study and performed the statistical analysis. DS, FM and PPDA contributed sending some scleroderma patients, essential for the study. MAC performed right heart catheterisation. DO, DA and TG participated in its design and coordination.

All authors read and approved the final manuscript.

## Consent

All patients provided written informed consent to participate in the research

## Supplementary Material

Additional file 1 **Patient's data**. Seventeen of the 19 patients had limited cutaneous systemic sclerosis, and two an overlap syndrome including scleroderma. Click here for file

Additional file 2 **ECHO an RHC data**. There was a significant correlation between TAPSE and NYHA class (r = -0.47; p > 0.05) and a good correlation between ACTpo and NYHA class (r = - 0.71; p > 0.001). Right heart catheterisation detected PAH in 15/19 patients: PAPm was 30.5 mmHg and PVR 3.6 UW. Click here for file
