# Supplementary figures and images for: Echocardiography may help detect pulmonary vasculopathy in the early stages of pulmonary artery hypertension associated with systemic sclerosis
Source: Cardiovasc Ultrasound. 2010 Jul 5;8:25. doi: 10.1186/1476-7120-8-25 (PMC2908574; doi:10.1186/1476-7120-8-25)

## Slide 1
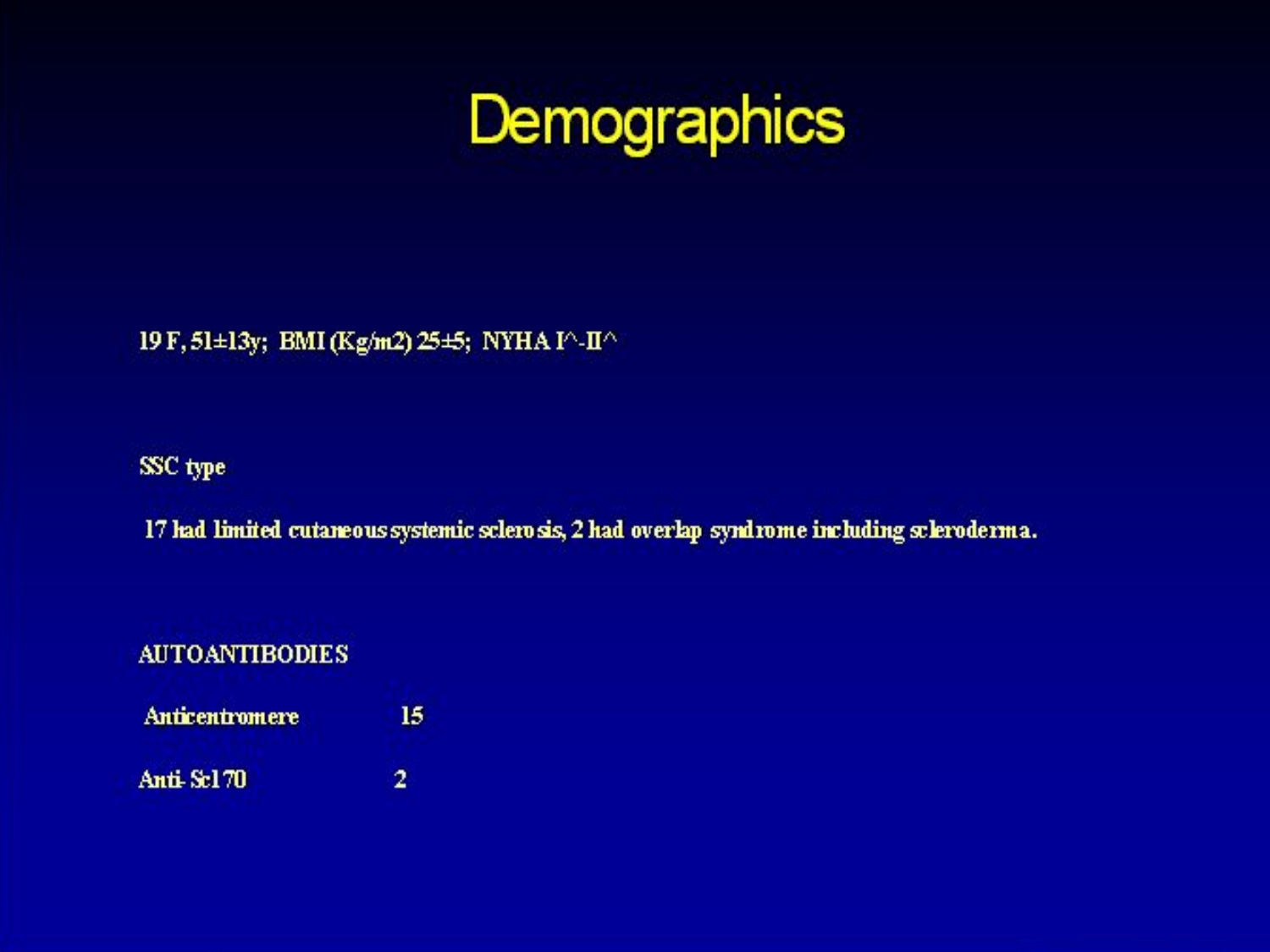

Supplement: Additional file 1 — Patient's data. Seventeen of the 19 patients had limited cutaneous systemic sclerosis, and two an overlap syndrome including scleroderma. [file 1476-7120-8-25-S1.PPT]

## Slide 1
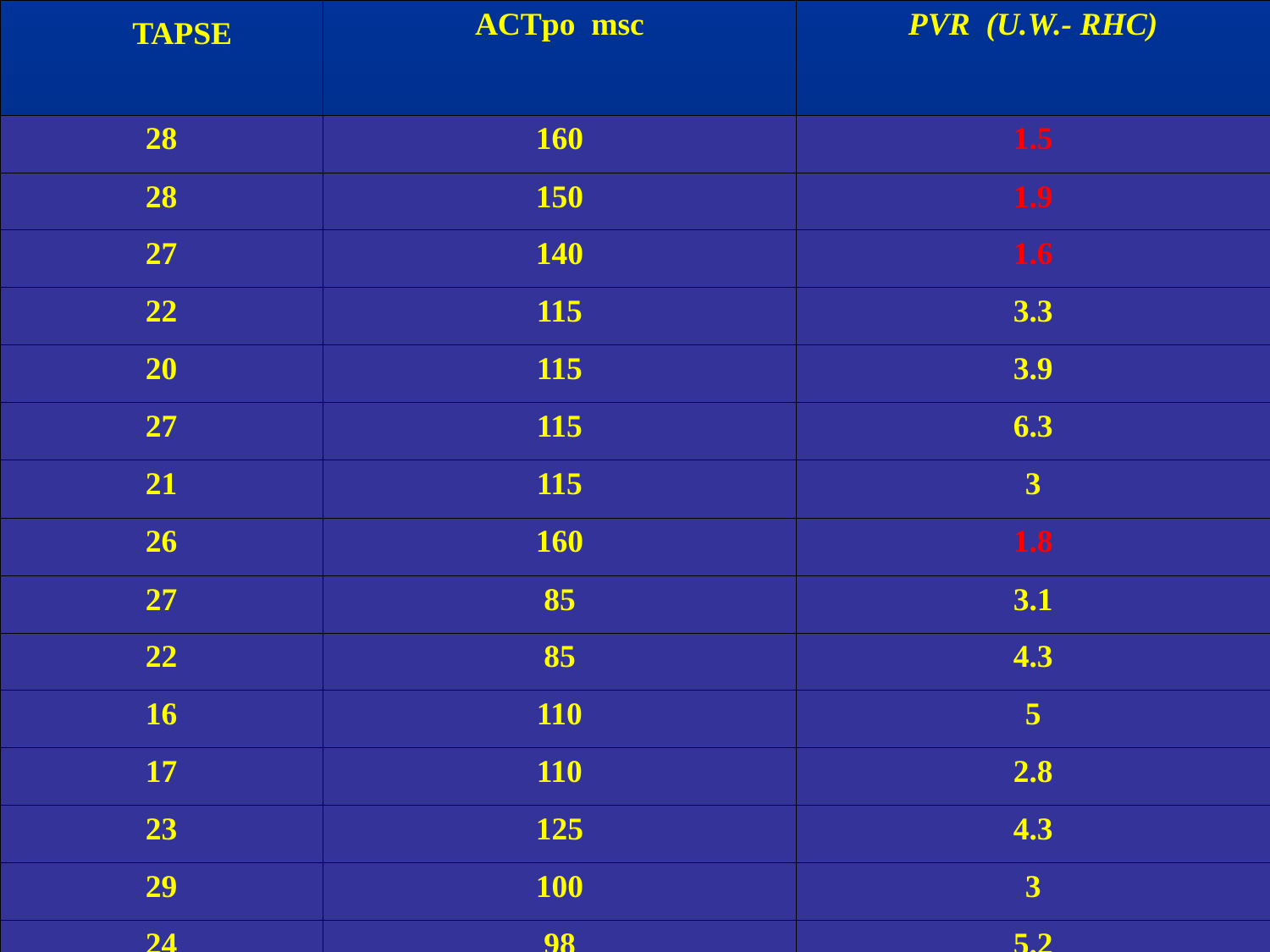

| TAPSE | ACTpo msc | PVR (U.W.- RHC) |
| --- | --- | --- |
| 28 | 160 | 1.5 |
| 28 | 150 | 1.9 |
| 27 | 140 | 1.6 |
| 22 | 115 | 3.3 |
| 20 | 115 | 3.9 |
| 27 | 115 | 6.3 |
| 21 | 115 | 3 |
| 26 | 160 | 1.8 |
| 27 | 85 | 3.1 |
| 22 | 85 | 4.3 |
| 16 | 110 | 5 |
| 17 | 110 | 2.8 |
| 23 | 125 | 4.3 |
| 29 | 100 | 3 |
| 24 | 98 | 5.2 |
| 21 | 120 | 2.8 |
| 22 | 135 | 2,8 |
| 27 | 120 | 3.5 |
| 20 | 128 | 2.8 |

Supplement: Additional file 2 — ECHO an RHC data. There was a significant correlation between TAPSE and NYHA class (r = -0.47; p > 0.05) and a good correlation between ACTpo and NYHA class (r = - 0.71; p > 0.001). Right heart catheterisation detected PAH in 15/19 patients: PAPm was 30.5 mmHg and PVR 3.6 UW. [file 1476-7120-8-25-S2.PPT]
